# Cardiorenal risk stratification in high-risk type 2 diabetes using a simple clinical score: findings from the ELIXA trial

**DOI:** 10.3389/fendo.2026.1768816

**Published:** 2026-03-18

**Authors:** Wenhui Jiang, Jingyu Wang, Jing Li, Yongmei Li, Yi Zhang, Jie Xu, Jinghang Zhang, Zhongai Gao, Jingli Cheng, Juhong Yang, Baocheng Chang

**Affiliations:** 1 NHC Key Lab of Hormones and Development and Tianjin Key Lab of Metabolic Diseases, Tianjin Medical University Chu Hsien-I Memorial Hospital & Institute of Endocrinology, Tianjin, China; 2 Cangzhou Hospital of Integrated Traditional Chinese and Western Medicine of Hebei Province, Cangzhou, China; 3 Guangdong Provincial Key Laboratory of Autophagy and Major Chronic Non-communicable Diseases, Key Laboratory of Prevention and Management of Chronic Kidney Disease of Zhanjiang, Institute of Nephrology, Endocrinology Department of Affiliated Hospital of Guangdong Medical University, Zhanjiang, China

**Keywords:** cardiorenal disease, cardiovascular diseases, cardiovascular-kidney-metabolic syndrome, diabetic kidney disease, risk assessment, type 2 diabetes mellitus

## Abstract

**Introduction:**

Early identification of patients with high cardiorenal risk and timely targeted interventions are critical in managing type 2 diabetes (T2D). Although we previously developed a multivariable risk score to predict diabetic kidney disease (DKD), its ability to stratify cardiorenal risk in T2D patients with established high cardiovascular risk remains unknown.

**Methods:**

In this *post-hoc* analysis of the ELIXA trial, 2,635 T2D participants without baseline DKD were stratified into different risk groups (low-, moderate-, high-, and very high-risk group) using the previously developed risk score (incorporating nine simple clinical indicators: age, body mass index, hemoglobin A1c, systolic blood pressure, high-density lipoprotein cholesterol, triglycerides, smoking, diabetic retinopathy, and urinary albumin-to-creatinine ratio [UACR]). Patients were followed for renal outcomes (DKD incidence and progression) and cardiovascular outcomes (major adverse cardiovascular events [MACEs] and heart failure [HF]).

**Results:**

The risk score demonstrated strong, graded associations with cardiorenal outcomes. Over 108 weeks of renal follow-up, a progressive increase in DKD incidence was observed across risk strata (52.5% vs. 13.5% in the very high- vs. low-risk groups; relative risks [RR] 3.89, 95% confidence interval [CI] 2.82**–**5.38, *P* < 0.001). This graded pattern extended to other key events of renal disease progression: macroalbuminuria (5.1% vs. 0.8%; RR 9.37, 95% CI 2.41**–**36.46, *P* = 0.001), a ≥40% decline in estimated glomerular filtration rate (eGFR, 3.4% vs. 0.2%; RR 17.72, 95% CI 1.58**–**198.82, *P* = 0.020), and rapid renal function decline (44.2% vs. 29.5%; RR 1.89, 95% CI 1.06**–**3.37; *P* = 0.032). Higher-risk groups exhibited earlier and progressively worsening renal dysfunction, with eGFR decline evident as early as week 24 and UACR elevation becoming significant by week 76, both persisting through week 108. During the 224-week cardiovascular follow-up, the combined high/very high-risk group had significantly greater risks of both MACEs (15.2% vs. 6.8%; hazard ratio [HR] 1.86, 95% CI 1.26**–**2.74, *P* = 0.001) and HF (2.8% vs. 0.5%; HR 4.58, 95% CI 1.41**–**14.9, *P* = 0.011) compared to the low-risk group.

**Discussion:**

This practical risk score identifies high-risk T2D patients with cardiorenal risk, including early renal function decline, to guide targeted intervention.

## 1 Introduction

As the global burden of diabetes continues to grow, diabetic kidney disease (DKD) has emerged as the predominant etiology of chronic kidney disease (CKD) worldwide (1). Beyond its role in driving progression to end-stage renal disease (ESRD), DKD also confers a markedly increased risk of cardiovascular complications and early death (2, 3). This interrelated pathophysiology is increasingly conceptualized under the cardiovascular–kidney–metabolic (CKM) syndrome paradigm (4, 5).

Although several pharmacotherapies have demonstrated efficacy in mitigating these interconnected CKM risks (6–8), a significant challenge remains in translating this evidence into routine practice, where their use is often delayed and inadequate (9, 10). Therefore, systematic risk assessment becomes a key tool to enable a targeted treatment approach, ensuring that high-risk patients are identified early and receive the full benefit of these essential therapies. Specifically, a tool capable of early, integrated cardiorenal risk stratification could enable timely initiation of organ-protective therapies, potentially altering the clinical trajectory in this high-risk population.

To address this need, our team previously developed a multivariable risk score for the early detection of DKD. This model was derived from multicenter cohorts of 41,000 patients with type 2 diabetes (T2D), incorporating key predictors identified through a systematic review and meta-analysis (11). The model integrates nine routinely available variables, encompassing characteristics (age, smoking status), key metabolic parameters (body mass index [BMI], systolic blood pressure [SBP], hemoglobin A1c [HbA1c], high-density lipoprotein cholesterol [HDL-C], triglycerides [TG]), and microvascular injury indicators (urinary albumin to creatinine ratio [UACR] and presence of diabetic retinopathy [DR]) (11). Its strong discriminatory power for DKD risk stratification has been confirmed in external validation cohorts (11–13).

A critical clinical gap persists in the early identification of patients at high integrated cardiorenal risk. Current tools are either siloed by organ system (cardiovascular vs. renal) or applicable only to late-stage disease (14, 15), failing to provide a unified assessment for patients with preserved kidney function who stand to benefit most from early CKM-targeted therapy.

To address this gap, this study aimed to evaluate the prognostic performance of this risk score in patients with both diabetes and elevated cardiovascular risk (a core CKM population). Moreover, the risk factors incorporated into the score align with the shared pathophysiological drivers of CKM syndrome (16). We therefore hypothesized that this risk score might possess a broader prognostic utility, extending its association to major cardiorenal endpoints beyond its original design.

Accordingly, we conducted this *post hoc* analysis of the Evaluation of Lixisenatide in Acute Coronary Syndrome (ELIXA) trial (17). The primary aim was to validate the integrated risk-stratification capacity of this DKD risk score in a high-risk cohort of patients with type 2 diabetes and established cardiovascular disease. Specifically, we sought to determine, first, whether the score stratifies risk for clinical cardiorenal endpoints (including DKD incidence, DKD progression, major adverse cardiovascular events [MACEs], and heart failure [HF] hospitalization) and, second, whether it identifies diverging early trajectories of renal function, as measured by longitudinal changes in UACR and estimated glomerular filtration rate (eGFR).

## 2 Materials and methods

### 2.1 Study hypothesis

We hypothesized that the multivariable risk score, comprised of factors representing shared CKM pathophysiology, would demonstrate a strong, graded association with a spectrum of cardiorenal outcomes, effectively stratifying risk in this high-risk population.

### 2.2 Study design and population

Our analysis utilized data from the ELIXA trial, a randomized, double-blind, placebo-controlled study conducted at 828 clinical sites across 49 countries between July 2010 and August 2013 (ClinicalTrials.gov identifier: NCT01147250) (17). It enrolled 6,068 adults aged ≥30 years with T2D (HbA1c 5.5%–11.0% [37–97 mmol/mol]) who had experienced an acute coronary syndrome (ACS) within 180 days (18). Eligible patients on stable glucose-lowering therapy for ≥12 weeks were randomized 1:1 to lixisenatide or placebo (17). The study protocol was approved by local ethics committees and institutional review boards, conducted in accordance with the Declaration of Helsinki and ICH guidelines, and all participants provided written informed consent (17).

We restricted our *post-hoc* analysis of the ELIXA trial to participants with normoalbuminuria and preserved kidney function at baseline, defined as UACR <30 mg/g and eGFR ≥60 mL/min/1.73 m², in accordance with KDIGO criteria (19). As the multivariable risk score was developed and validated in White and Asian populations aged 39 to 75 years, we excluded individuals outside the age range of 39–75 years and those who were not of White or Asian race. We also excluded individuals with unknown diabetic retinopathy status or missing values for BMI, HbA1c, HDL-C, SBP, TG, or UACR. In total, 8 participants (0.13% of the screened population) were excluded on this basis.

The multivariable risk prediction score, originally developed by our group, incorporates nine baseline variables (11). Points are assigned based on predefined risk categories as follows: age 39–49, 50–59, and 60–75 years receive 0, 3.0, and 6.0 points, respectively; non-smokers receive 0 points and current smokers 4.0 points; BMI <25.00, 25.00–29.99, and ≥30.00 kg/m² are assigned 0, 1.5, and 3.0 points; SBP <130, 130–139, 140–149, and ≥150 mmHg are scored 0, 2.0, 4.0, and 6.0 points; HbA1c <7.0% (<53 mmol/mol), 7.0–7.9% (53–63 mmol/mol), 8.0–8.9% (64–74 mmol/mol), and ≥9.0% (≥75 mmol/mol) correspond to 0, 1.5, 3.0, and 4.5 points, respectively; HDL-C ≥1.3 mmol/L receives 0 points and <1.3 mmol/L 2.5 points; TG <1.7 mmol/L are assigned 0 points and ≥1.7 mmol/L 4.0 points; absence of DR is scored 0 and presence 3.0 points; and UACR <10.00, 10.00–19.99, and 20.00–29.99 mg/g receive 0, 2.0, and 4.0 points, respectively (11). The risk score was applied to baseline data from eligible ELIXA participants, and individuals were categorized into four risk groups based on their total scores: low (<12.0), moderate (12.0–15.5), high (16.0–26.5), and very high (27.0–37.0), according to the risk stratification scheme established in our prior multicenter cohort study (11).

### 2.3 Study outcomes

The study assessed renal and cardiovascular outcomes over different follow-up periods. Renal outcomes were assessed over 108 weeks. Serum creatinine and two morning urine samples were collected at baseline, weeks 24, 76, and 108, and UACR was calculated as the mean of two samples obtained on the day before and the day of each clinic visit, in accordance with the standardized procedures of the ELIXA trial (17). Applying the Chronic Kidney Disease Epidemiology Collaboration (CKD-EPI) equation (20), we derived the values for eGFR. Cardiovascular outcomes were assessed over a longer follow-up period of up to 224 weeks.

### 2.4 Definition of study outcomes

#### 2.4.1 Renal outcomes

Incident DKD was defined as the development of an eGFR < 60 mL/min/1.73 m² and/or a UACR ≥ 30 mg/g, in the absence of other primary or secondary kidney diseases, according to the current Kidney Disease: Improving Global Outcomes (KDIGO) guidelines (19).
DKD progression was assessed using the following criteria: UACR progression: (1) UACR progression, defined as a ≥40% increase from baseline or progression to macroalbuminuria (UACR ≥300 mg/g) (21); (2) eGFR decline, defined as a ≥40% reduction from baseline, as recommended by the National Kidney Foundation and the U.S. Food and Drug Administration (22); and (3) rapid eGFR decline, defined as an annualized eGFR decline of ≥3.3 mL/min/1.73 m²/year, as recommended by the National Kidney Foundation and the U.S. Food and Drug Administration (23). The annualized rate of eGFR decline was calculated as (baseline eGFR – eGFR at week 108)/follow-up time (years).

#### 2.4.2 Cardiovascular outcomes

We evaluated two primary cardiovascular endpoints: (1) MACEs, defined as nonfatal myocardial infarction, nonfatal stroke, hospitalization for unstable angina, or cardiovascular death, as prespecified in the ELIXA trial protocol (17); and (2) hospitalization due to heart failure (HF).

### 2.5 Statistical analysis

For baseline summary, categorical and continuous variables are presented as percentages (%) and means ± standard deviations, respectively. Continuous variables with a skewed distribution are summarized as medians with interquartile ranges (IQRs). Group comparisons were performed using the Chi-square test for categorical variables and one-way ANOVA for continuous variables. We calculated the incidences of both renal and cardiovascular outcomes across the risk groups. For renal outcomes, we used Generalized Estimating Equations (GEE) to derive relative risks (RRs) with 95% confidence intervals [CIs]. The moderate-, high-, and very high-risk groups were compared against the low-risk group as the reference. Using a mixed-effects model for repeated measures (MMRM), we assessed the longitudinal trajectories of mean eGFR and UACR and tested for overall between-group differences over time. The percentage changes in eGFR and UACR from baseline to week 24, 76 and 108 across risk subgroups were assessed using MMRM with risk subgroup, visit, and risk subgroup-by-visit interactions in the model. Least-squares (LS) means (standard error, SE) for each risk group and LS mean differences (SE) between risk levels with *P* values were presented for all follow-up visits. To address the marked skewness in UACR data, values were log-transformed for analysis to approximate normality, and the results were back-transformed to express changes from baseline to week 108 as percentages derived from geometric means and their standard errors. For the analysis of cardiovascular events, we merged the high-risk and very high-risk groups into a combined ‘high/very high-risk’ group. Kaplan-Meier curves were utilized to assess the development of MACEs and HF according to different risk groups, with significant differences between groups assessed using a log-rank test. Cox proportional hazards analysis was used to compute hazard ratios (HRs) and 95% CIs to assess different risk groups (moderate- and high/very high-risk group) on MACEs and HF, with the low risk-group used as a reference. A two-sided *P* value of less than 0.05 was considered indicative of statistical significance for all analyses. Statistical analyses were performed using SAS Enterprise Guide software (version 7.13; SAS Institute Inc., Cary, NC, USA).

## 3 Results

### 3.1 Study population and baseline characteristics

**Figure 1:**
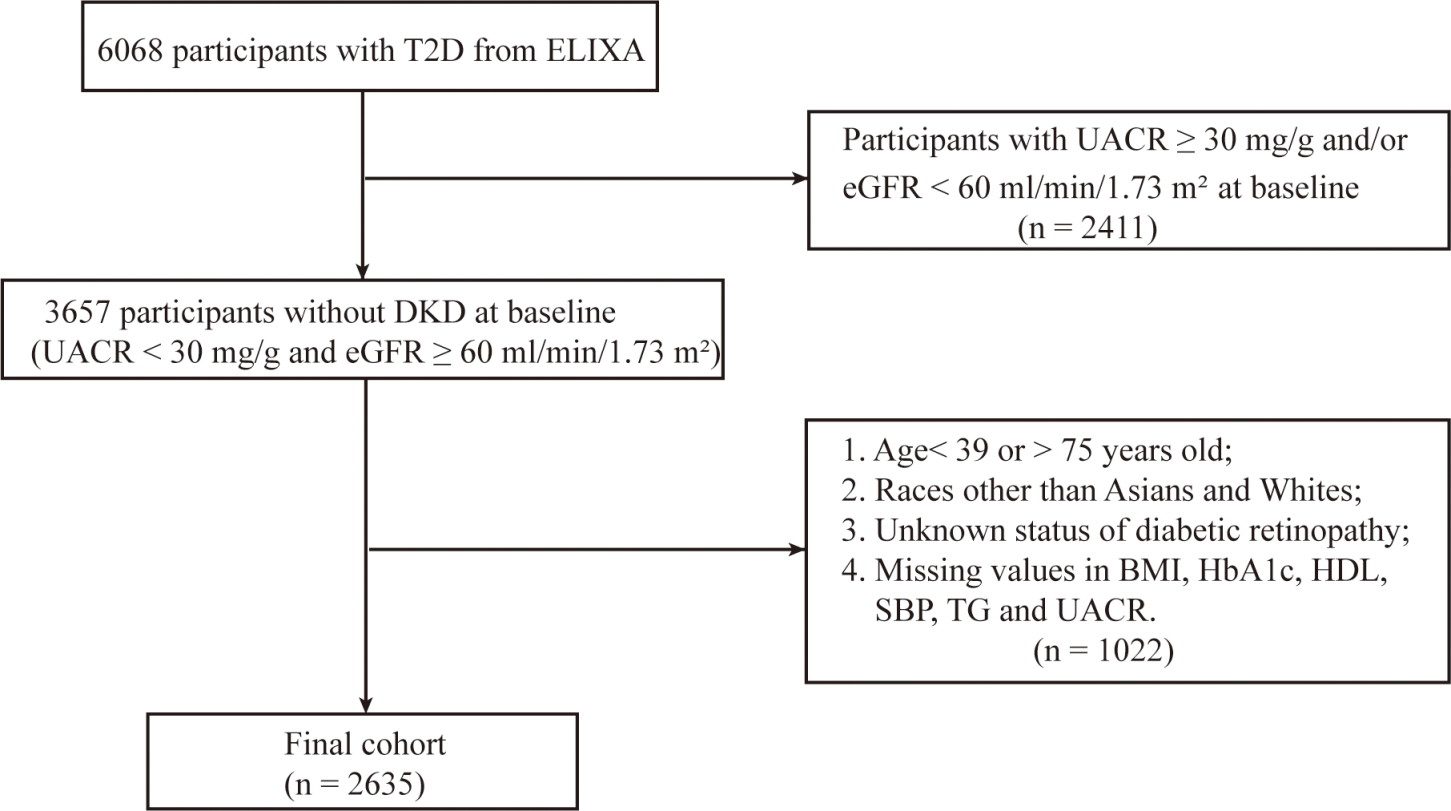
Flow chart of participant selection.

The study cohort comprised 2,635 ELIXA participants meeting the baseline criteria of UACR < 30 mg/g and eGFR ≥ 60 mL/min/1.73 m². The flowchart demonstrated the distribution of the study participants (**Figure 1**). The cohort was divided into four risk groups according to baseline DKD risk scores: low (n = 533), moderate (n = 750), high (n = 1,293), and very high (n = 59). This population presented with typical baseline characteristics: a mean age of 58.24 years (± 8.08) and a diabetes duration of 7.82 years (± 7.24). Their metabolic profiles included a BMI of 30.15 kg/m² (± 5.48) and an HbA1c of 7.59% (± 1.28). Renal assessments revealed a mean UACR of 7.4 mg/g and an eGFR of 84.29 mL/min/1.73 m². Baseline characteristics were summarized in **Table 1**.

**Table 1:** Baseline characteristics of patients by different risk groups according to DKD risk score.

Characteristics	ALL (n = 2635)	Risk groups				
		Low (n = 533)	Moderate (n = 750)	High (n = 1293)	Very high (n = 59)	*P* value
Age (years)	58.24 ± 8.08	54.10 ± 8.02	57.67 ± 8.05	60.03 ± 7.45	64.00 ± 6.08	<0.0001
Male, n (%)	1953 (74.1)	400 (75.0)	565 (75.3)	944 (73.0)	44 (74.6)	0.6474
Diabetes Duration (years)	7.82 ± 7.24	5.87 ± 6.08	7.26 ± 6.70	8.73 ± 7.62	12.38 ± 9.26	<0.0001
Systolic BP (mmHg)	127.61 ± 15.92	118.49 ± 11.25	123.67 ± 13.60	132.76 ± 16.15	146.86 ± 14.62	<0.0001
Diastolic BP (mmHg)	77.24 ± 9.57	74.11 ± 8.18	76.35 ± 9.24	78.68 ± 9.73	85.00 ± 11.24	<0.0001
BMI (kg/m^2^)	30.15 ± 5.48	28.39 ± 5.43	29.69 ± 5.33	31.06 ± 5.41	31.97 ± 4.36	<0.0001
HDL-C (mmol/L)	1.11 ± 0.27	1.18 ± 0.30	1.13 ± 0.27	1.06 ± 0.25	1.06 ± 0.17	<0.0001
LDL-C (mmol/L)	2.01 ± 0.89	1.89 ± 0.78	1.97 ± 0.89	2.08 ± 0.94	2.11 ± 0.79	<0.001
Triglycerides (mmol/L)	1.78 ± 1.19	1.27 ± 0.58	1.58 ± 1.06	2.08 ± 1.34	2.45 ± 1.10	<0.0001
HbA1c (%)	7.59 ± 1.28	7.02 ± 1.08	7.38 ± 1.23	7.89 ± 1.27	8.70 ± 1.18	<0.0001
FPG (mmol/L)	8.18 ± 2.71	7.29 ± 2.13	7.78 ± 2.54	8.69 ± 2.83	10.35 ± 3.31	<0.0001
eGFR (mL/min/1.73 m^2^)	84.29 ± 16.31	85.20 ± 14.97	84.59 ± 15.80	83.86 ± 17.09	81.46 ± 16.52	0.0172
UACR (mg/g)	7.4 (5.1, 11.5)	5.9 (4.4, 8.3)	6.6 (4.7, 9.5)	8.8 (5.8, 14.1)	15.8 (11.2, 22.6)	<0.0001
Retinopathy, n (%)	226 (8.6)	7 (1.3)	35 (4.7)	157 (12.1)	27 (45.8)	<0.0001
Current smoker, n (%)	325 (12.3)	32 (6.0)	76 (10.1)	210 (16.2)	7 (11.9)	<0.0001
Race, n (%)						<0.0001
Asian	446 (16.9)	134 (25.1)	147 (19.6)	159 (12.3)	6 (10.2)	
White	2189 (83.1)	399 (74.9)	603 (80.4)	1134 (87.7)	53 (89.8)	
ACE-I, n (%)	1645 (62.4)	328 (61.5)	482 (64.3)	801 (61.9)	34 (57.6)	0.5793
ARB, n (%)	584 (22.2)	107 (20.1)	148 (19.7)	311 (24.1)	18 (30.5)	0.0297
Statins, n (%)	2456 (93.2)	502 (94.2)	708 (94.4)	1191 (92.1)	55 (93.2)	<0.0001

Data are presented as n (%), mean ± SD, or median (quartiles). DKD, diabetic kidney disease; BP, blood pressure; BMI, body mass index; HDL-C, high-density lipoprotein cholesterol; LDL-C, low-density lipoprotein cholesterol; HbA1c, hemoglobin A1c; FPG, fasting plasma glucose**;** eGFR, estimated glomerular filtration rate; UACR, urinary albumin-to-creatinine ratio; ACE-I, angiotensin-converting enzyme inhibitor; ARB, angiotensin receptor blocker; n, number of patients.

### 3.2 Incidence of DKD by risk groups

**Figure 2:**
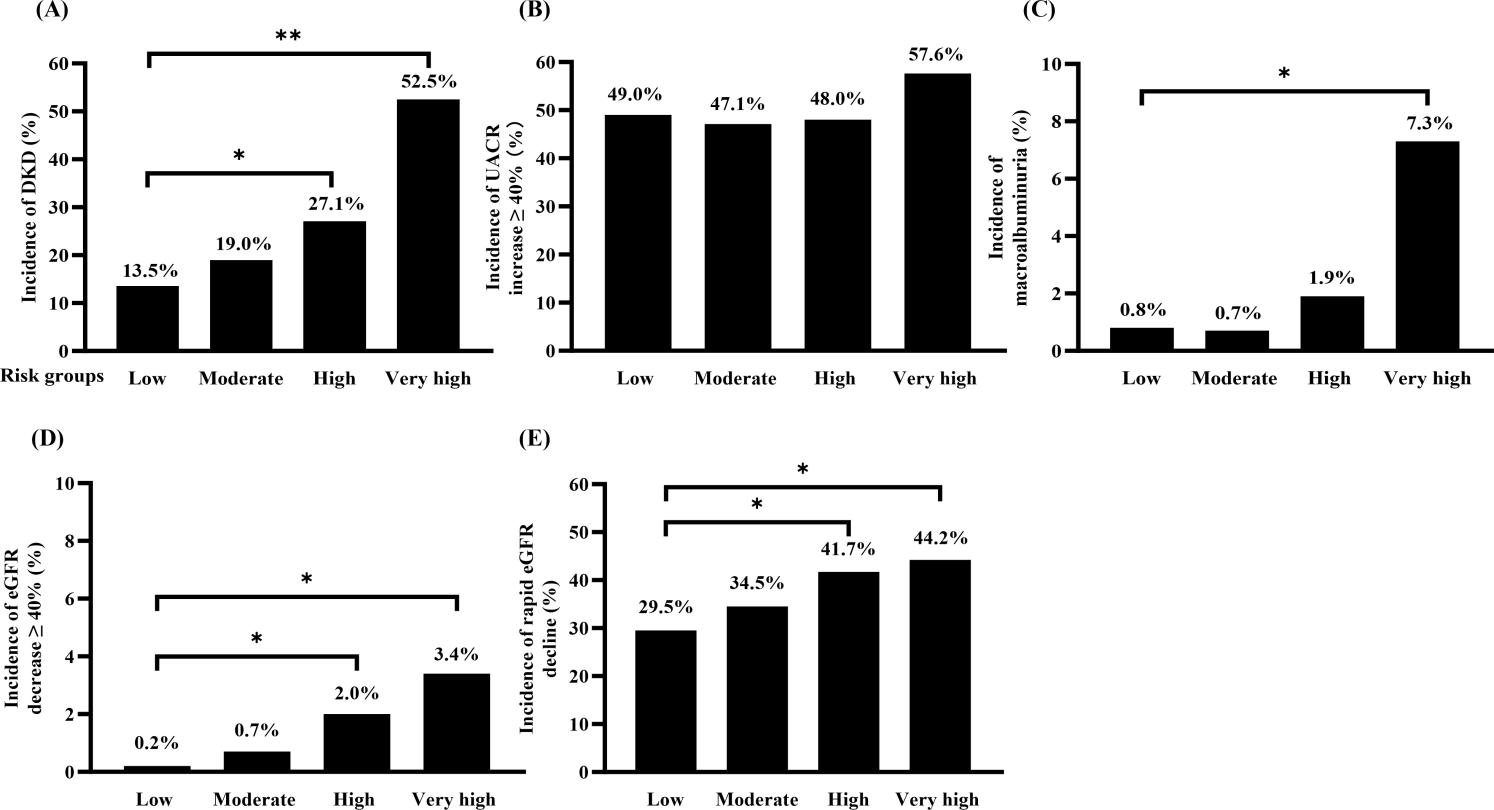
Incidences of **(A)** DKD development, and **(B)** UACR increment ≥ 40%, and **(C)** macroalbuminuria, and **(D)** eGFR decline ≥40%, and **(E)** rapid eGFR decline in different risk groups. *P < 0.05; **P < 0.001. DKD, diabetic kidney disease; UACR, urinary albumin-to-creatinine ratio; eGFR, estimated glomerular filtration rate.

Over 108 weeks, DKD developed in 595 patients (22.6%), with incidence rates sharply escalating with increasing risk scores: 13.5% (low-risk), 19.0% (moderate-risk), 27.1% (high-risk), and 52.5% (very high-risk) (**Figure 2A**). Corresponding to this gradient, the RRs for DKD development, compared to the low-risk group, were 1.40, 2.00, and 3.89 for the moderate-, high-, and very high-risk groups, respectively (all *P* < 0.05) (**Table 2**).

**Table 2:** Incidences and relative risks of kidney outcomes by different risk groups according to DKD risk score.

Kidney outcomes	Risk groups			
	Low	Moderate	High	Very high
DKD development				
n/N (%)	72/533 (13.5)	142/749 (19.0)	350/1293 (27.1)	31/59 (52.5)
RR (95% CI)	reference	1.40 (1.08, 1.82)	2.00 (1.59, 2.53)	3.89 (2.82, 5.38)
*P* value		0.011	< 0.001	< 0.001
UACR increase ≥ 40%				
n/N (%)	261/533 (49.0)	353/749 (47.1)	620/1293 (48.0)	34/59 (57.6)
RR (95% CI)	reference	0.96 (0.86, 1.08)	0.98 (0.88, 1.08)	1.18 (0.93, 1.49)
*P* value		0.508	0.673	0.169
Macroalbuminuria				
n/N (%)	4/506 (0.8)	5/724 (0.7)	23/1223 (1.9)	4/55 (7.3)
RR (95% CI)	reference	0.87 (0.24, 3.24)	2.38 (0.83, 6.84)	9.37 (2.41, 36.46)
*P* value		0.839	0.108	0.001
eGFR decline ≥ 40%				
n/N (%)	1/533 (0.2)	5/749 (0.7)	26/1293 (2.0)	2/59 (3.4)
RR (95% CI)	reference	3.60 (0.42, 30.88)	11.05 (1.50, 81.66)	17.72 (1.58, 198.82)
*P* value		0.243	0.019	0.020
Rapid eGFR decline				
n/N (%)	147/498 (29.5)	246/714 (34.5)	501/1202 (41.7)	23/52 (44.2)
RR (95% CI)	reference	1.26 (0.98, 1.61)	1.71 (1.36, 2.14)	1.89 (1.06, 3.37)
*P* value		0.071	< 0.0001	0.032

DKD, diabetic kidney disease; UACR, urinary albumin-to-creatinine ratio; eGFR, estimated glomerular filtration rate; n, number of events; N, total number of subjects in the corresponding risk group; RR, relative risk; CI, confidence interval.

### 3.3 Progression of DKD by risk groups

The risk score effectively stratified patients for the risk of DKD progression, with significant gradients observed for the development of macroalbuminuria and eGFR decline, but not for a ≥40% increase in UACR.

The proportion of patients who developed macroalbuminuria increased progressively across risk strata, from 0.8% in the low-risk group to 7.3% in the very high-risk group (**Figure 2C**). Compared to the low-risk group, only the very high-risk group showed a significantly increased relative risk (RR 9.37, 95% CI 2.41–36.46; *P* = 0.0012) (**Table 2**). In contrast, the risk of a ≥40% increase in UACR did not differ significantly among the groups.

A strong, graded association was observed between the risk score and measures of renal function decline. The incidence of a ≥40% decline in eGFR rose from 0.2% in the low-risk group to 3.4% in the very high-risk group (**Figure 2D**), corresponding to significantly elevated RRs of 11.05 and 17.72 in the high- and very high-risk groups, respectively. Similarly, the proportion of patients with rapid eGFR decline increased stepwise from 29.5% to 44.2% across the risk spectrum (**Figure 2E**), with progressively higher RRs of 1.41 and 1.50 in the high- and very high-risk groups (**Table 2**).

### 3.4 Longitudinal changes in renal function across risk groups

**Figure 3:**
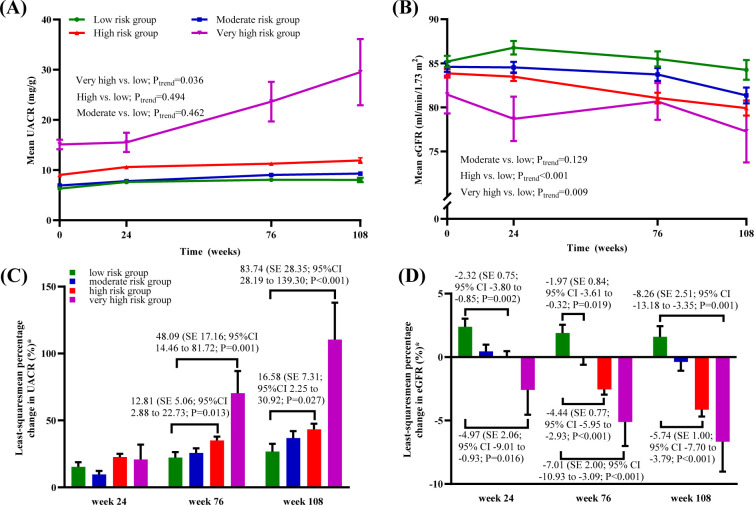
Longitudinal trajectories of UACR and eGFR. **(A)** Mean UACR trajectory**;**and **(B)** Mean eGFR trajectory; **(C)** Least-squares mean percentage change in UACR, by baseline albuminuria status; **(D)** Least-squares mean percentage change in eGFR, by baseline eGFR status. **(A, B)**. Geometric mean values and SEs are shown. **(C, D).** Data are geometric means. Error bars show SEs. Data represent least-squares mean differences (SEs) for moderate, high or very high-risk groups versus low risk-group, and the corresponding 95% CIs and *P* values. Abbreviations: UACR, urinary albumin-to-creatinine ratio; eGFR, estimated glomerular filtration rate.

The longitudinal assessment revealed that renal function trajectories diverged early across risk groups. For UACR, a significant increasing trend was observed in the very high–risk group (*P*_trend_ = 0.036). By week 76, LS mean percentage changes from baseline were significantly higher in the high- and very high-risk groups compared with the low-risk group, with estimated increases of 12.81% (SE = 5.06; 95% CI 2.88–22.73; *P* = 0.013) and 48.09% (SE = 17.16; 95% CI 14.46–81.72; *P* = 0.001), respectively (**Figures 3A, C**). For eGFR, significant declining trends versus the low–risk group were evident in both the high–risk (*P*_trend_ < 0.001) and very high–risk groups (*P*_trend_ = 0.009). A significant declining trend of eGFR emerged as early as week 24 in the high-risk groups, with LS mean percentage changes of -2.32% (SE = 0.75; 95% CI -3.80 to -0.85; *P* = 0.002) in the high-risk group and -4.97% (SE = 2.06; 95% CI -9.01to -0.93; *P* = 0.016) in the very high-risk group. Between-group differences in both UACR elevation and eGFR decline persisted and continued to widen throughout the 108-week follow-up (**Figures 3C, D**).

### 3.5 Cardiovascular outcomes by risk group

**Figure 4:**
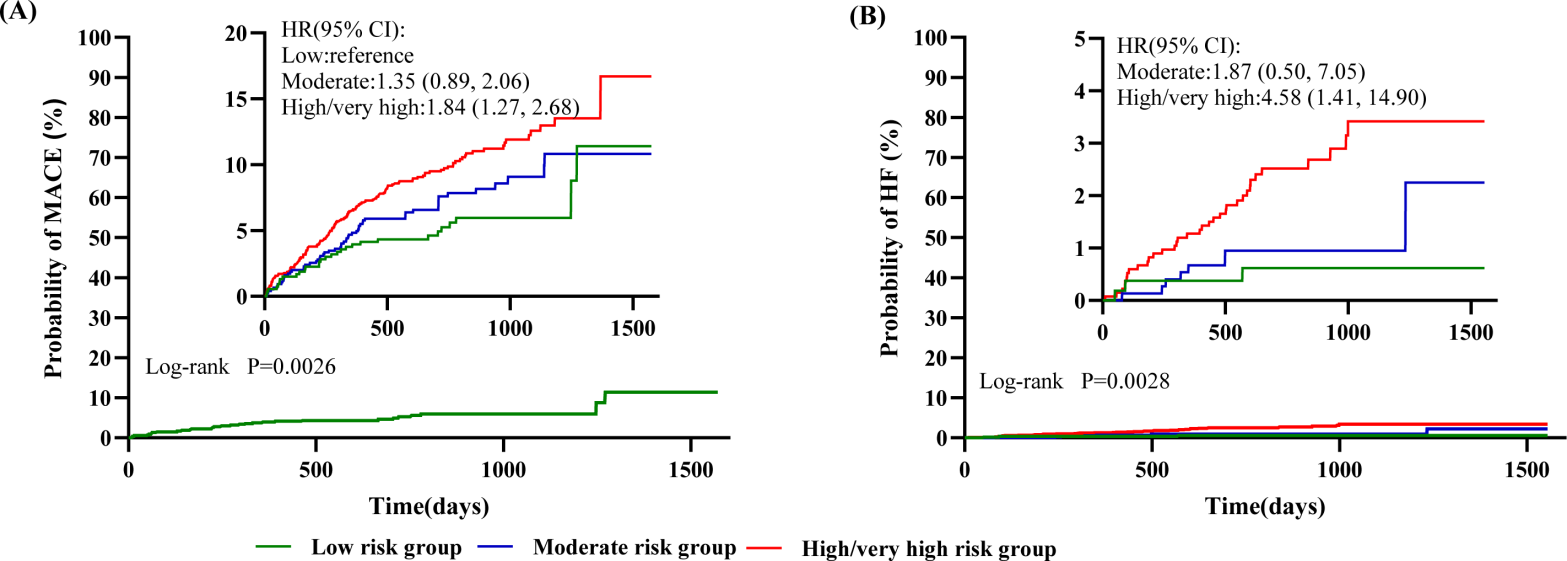
Kaplan–Meier curves of **(A)** major adverse cardiovascular events (MACEs) and; **(B)** heart failure (HF). The MACEs in the time-to-event analysis was death from cardiovascular causes, nonfatal myocardial infarction, nonfatal stroke, or hospitalization for unstable angina. The inset shows the same data on an enlarged y axis.

The incidences of MACEs and HF increased across the low-, moderate-, and high/very high-risk groups (**Table 3**). Consistent with this trend, survival curve analysis demonstrated that patients in the high/very high-risk group faced significantly elevated risks for both MACEs (HR, 1.86; 95% CI 1.26–2.74; *P* = 0.0017) and HF (HR, 4.58; 95% CI 1.41–14.9; *P* = 0.0113), as shown in **Figure 4**.

**Table 3:** Incidences and hazard ratios of major adverse cardiovascular events and heart failure across different risk groups.

Outcome	Risk groups		
	Low	Moderate	High/very high
MACEs			
n/N (%)	31/533 (5.8)	59/749 (7.9)	145/1352 (10.7)
HR (95% CI)	reference	1.35 (0.89, 2.06)	1.84 (1.27, 2.68)
*P* value	–	0.157	0.001
HF			
n/N (%)	3/533 (0.6)	8/749 (1.1)	35/1352 (2.6)
HR (95% CI)	reference	1.87 (0.50, 7.05)	4.58 (1.41, 14.90)
*P* value	–	0.355	0.011

n, number of events; N, total number of subjects; HR, hazard ratio; CI, confidence interval; MACEs, major adverse cardiovascular events; HF, heart failure.

## 4 Discussion

The principal finding of this study is that a multivariable risk score can identify, at baseline, distinct and diverging trajectories of cardiorenal risk in a high-risk diabetic population. We found a strong, graded association between the risk score and a spectrum of adverse outcomes, from the microvascular progression of kidney disease to hard cardiovascular events. Most importantly, the score could detect diverging trajectories of renal function early in the course of the study, characterized by a concurrent rise in albuminuria and a steeper decline in eGFR among high-risk patients.

Our findings are powerfully contextualized by the integrative paradigm of CKM syndrome, which posits that type 2 diabetes, CKD, and CVD are interconnected manifestations of shared pathophysiological pathways (5). The strength of our risk score lies in its integration of key, routinely measured cardiometabolic risk factors—specifically glycemic status (HbA1c), blood pressure (SBP), lipid profile (HDL-C, TG), and adiposity (BMI) (24–27)—into a single tool, which collectively represents the core pathophysiological drivers of cardiorenal risk 16. These factors collectively promote endothelial dysfunction, chronic inflammation, atherogenesis, thrombosis, myocardial injury, fibrosis, and chronic insulin resistance, significantly increasing the risk of vascular and kidney diseases, which are fundamental processes in the development of both kidney injury and atherosclerotic cardiovascular disease (28–30). Therefore, the score effectively quantifies an individual’s aggregate burden of CKM-related risk, thereby explaining its capacity to stratify patients for both renal and cardiovascular events.

Beyond the core metabolic factors, our model is further enriched by key demographic, behavioral, and early injury markers that compound cardiorenal risk. Age and smoking represent non-modifiable and modifiable risk factors, respectively, that accelerate shared pathways of vascular senescence and endothelial dysfunction (24, 31). Critically, the model also incorporates indicators of established markers of microvascular injury—specifically UACR for renal endothelial damage and DR for parallel microvascular pathology (11, 32–34). The inclusion of these diverse elements allows the multivariable risk score to capture a more comprehensive profile of an individual’s aggregate risk.

Building upon this pathophysiological foundation, our analysis indicates that the utility of this score extends beyond its original design objectives. It not only predicts the occurrence of DKD but, more importantly, identifies patients at high risk for DKD progression and cardiovascular events—associations that are being reported for the first time in a high-risk population. To understand this broader predictive capability, it is helpful to reconsider the score’s performance. The combination of modest discriminative performance—reflected by the area under the receiver operating characteristic curve—and highly significant relative risks suggests that the model functions less as a classifier for a specific outcome and more as a robust tool for stratifying patients along a continuum of cardiorenal risk.

Our risk score addresses a critical unmet need in the management of T2D: integrated cardiorenal risk assessment in patients who still have preserved renal function and normoalbuminuria. Existing tools are either focused exclusively on atherosclerotic cardiovascular disease [e.g., PREVENT equations (14, 15)] or designed for patients with established chronic kidney disease (CKD), such as the Kidney Failure Risk Equation (KFRE) (35), KDpredict (36), and Z6 Score (37), which predict hard renal outcomes in advanced CKD. Similarly, models validated in populations with severely reduced eGFR (38) are not applicable to early-stage disease. In contrast, our risk score captures distinct gradients of cardiorenal risk within this secondary prevention cohort—integrating modifiable metabolic factors and subclinical injury markers (UACR and DR). It thus identifies, among patients with established cardiovascular disease, those at highest risk for subsequent cardiorenal complications. This stratification remains clinically actionable: patients with elevated scores are likely to derive greater absolute benefit from intensification of cardiorenal-protective therapies [e.g., sodium–glucose cotransporter-2 inhibitors or glucagon-like peptide-1 receptor agonists (39)], even before the onset of overt albuminuria or a decline in eGFR.

Our risk score demonstrates clinical value at three distinct levels. First, it integrates core pathophysiological elements of CKM—modifiable risk factors including dysglycemia, hypertension, dyslipidemia, and adiposity—to create a tool capable of simultaneously assessing cardiorenal comorbidity risk. Second, and most importantly, we found that patients identified as high-risk by this model exhibited features of accelerated renal function deterioration early in the follow-up period (within 24 weeks). This confirms that the score not only identifies a high-risk status but, more critically, pinpoints those patients who have already entered a clinically observable trajectory of rapid renal function decline. Third, the risk score relies entirely on routine outpatient measurements and is simple to calculate. Although its variable weights were originally derived from a DKD prediction model, their basis in shared risk factors for cardiorenal disease naturally affords the score clinical interpretability and enables immediate implementation.

Despite these strengths, several limitations warrant consideration. First, this risk score was established based on risk factors for DKD development, rather than being specifically designed to predict cardiorenal events. Several potentially influential factors—such as genetic background, ethnicity, other adverse lifestyle factors, and B-type natriuretic peptide—were not incorporated into the model. Second, this analysis was *post hoc*, and the participants experienced ACS recently. There is a lack of validation for cardiorenal prognosis in patients with uncomplicated T2D (free of CV events). This approach, however, ensured a sufficient number of CV events within a relatively short follow-up period (lasting up to 224 weeks). Further validation in the general T2D population is warranted to elucidate its potential for CVD prediction. Third, for some outcome estimates—particularly those with high relative risks such as the ≥40% eGFR decline—the observed confidence intervals were notably wide (e.g., 1.58–198.82). This reflects limited precision, likely attributable to the relatively low absolute number of these specific endpoint events in our cohort. While these results strongly indicate a graded increase in risk, the exact magnitude of association remains uncertain. Future studies with larger sample sizes are needed to obtain more precise estimates. Fourth, our analysis employed a complete-case approach for the key model variables. Although the proportion of excluded participants was minimal (0.13%), potentially limiting the impact of selection bias, this methodological choice should be acknowledged. Future studies could employ advanced imputation techniques to confirm the robustness of our findings.

## 5 Conclusions

In conclusion, this study demonstrates that a simple multivariable risk score, derived from routinely available clinical parameters, effectively stratifies cardiorenal risk in high-risk individuals with T2D. It identifies patients not merely at risk for DKD onset, but those already on a trajectory of early renal function decline and at heightened risk for cardiovascular events. This integrated risk assessment, grounded in the CKM framework, provides a practical tool to guide early, targeted intervention with cardiorenal-protective therapies, thereby addressing a critical gap in the proactive management of this high-risk population.

## Data Availability

The data analyzed in this study is subject to the following licenses/restrictions: IPD from the underlying trial are not publicly available. Access is possible via Vivli upon approval by the sponsor (Sanofi) and is restricted to analysis within a secure environment. Inquiries may be directed to the corresponding author. Requests to access these datasets should be directed to https://vivli.org/.
